# *C. albicans* Colonization of Human Mucosal Surfaces

**DOI:** 10.1371/journal.pone.0002067

**Published:** 2008-04-30

**Authors:** Peter Southern, Julie Horbul, Diane Maher, Dana A. Davis

**Affiliations:** Department of Microbiology, University of Minnesota, Minneapolis, Minnesota, United States of America; Theodor-Boveri-Institut fur Biowissenschaften, Wurzburg, Germany

## Abstract

**Background:**

*Candida albicans* is a low level commensal organism in normal human populations with the continuous potential to expand and cause a spectrum of clinical conditions.

**Methodology/Principal Findings:**

Using *ex vivo* human organ cultures and populations of primary human cells, we have developed several related experimental systems to examine early-stage interactions between *C. albicans* and mucosal surfaces. Experiments have been conducted both with exogenously added *C. albicans* and with overtly normal human mucosal surfaces supporting pre-existing infections with natural isolates of Candida. Under different culture conditions, we have demonstrated the formation of *C. albicans* colonies on human target cells and filament formation, equivalent to tissue invasion.

**Conclusions/Significance:**

These organ culture systems provide a valuable new resource to examine the molecular and cellular basis for Candida colonization of human mucosal surfaces.

## Introduction

*Candida albicans* is widely distributed throughout the human population as both a commensal organism and as an intermittent pathogen. Limited numbers of viable organisms can be cultured from the oropharynx and from the vaginal cavity of normal individuals [1], [2]. However, as a consequence of microbial imbalance and/or transient immunosuppression, *C. albicans* overgrowth can readily occur to cause mucosal infections, including oral candidiasis (thrush) or vaginal candidiasis [3], [4], [5]. Thrush is frequently the first clinical symptom recognized in patients infected with human immunodeficiency virus type 1 (HIV-1) who experience declining CD4 T cell numbers prior to the onset of overt AIDS [6]. In the general population, vaginal candidiasis is a significant problem in sexually active young women, with millions of cases occurring annually in the USA [7], [8]. *C. albicans* can be regarded as a legitimate sexually transmitted infection (STI) because *C. albicans* sequences have been amplified by PCR using total DNA extracted from semen [9], [10]. Sexual practices involving oral-genital contacts may also account for spread and maintenance of Candida in sexually active populations [11]. In severely immunocompromised patients and in the recovery phase following major intestinal surgery, Candida can enter the bloodstream and cause life-threatening systemic infections [12].

*C. albicans* is a pleiomorphic fungus and the ability to transition between yeast cell forms and filamentous forms is essential for virulence and therefore constitutes a key component in *C. albicans* pathogenesis. Various models have been established to study Candida-epithelial cell interactions using immortalized human epithelial cell lines in tissue culture [13], [14], [15]. Experimental infections of laboratory mice have been used to identify *C. albicans* virulence factors and to identify elements of the immune system that confer protection against systemic Candida infection [16]. More recent studies have exploited invertebrate hosts to analyze fungal-host interactions [17]. However, none of these established systems provides an appropriate experimental resource to study primary *C. albicans* colonization and subsequent invasion of human mucosal surfaces and considerable effort has been invested to establish three-dimensional epithelial models [18], [19], [20], [21], [22], [23] . In this study, we have adapted human mucosal organ culture systems that were first developed to analyze primary HIV infection [24], [25] to devise a novel system that allows direct evaluation of *C. albicans* surface colonization and tissue invasion. Organ cultures based on human palatine tonsil and premenopausal cervical tissue utilize biologically relevant tissue surfaces to study the earliest events in Candida infections. Additionally, we have used primary populations of human tonsillar and cervical cells - both epithelial cells and fibroblasts [26] - to follow Candida colonization.

There are extensive structural similarities between the epithelial surfaces of the palatine tonsil and the cervix and comparative functional analyses will be highly informative in developing a comprehensive understanding of the primary events underlying *C. albicans* colonization. The surface of palatine tonsil is covered by a stratified squamous epithelium where the most external cells are continuously lost by sloughing and replaced by cells that are displaced upwards from the proliferating suprabasal cell layers. A similar stratified squamous epithelium protects the surface of the vagina and the ectocervix. The surface configuration of the tonsillar crypts is distinctly different from the multi-layered stratified epithelium covering most of the external surface of the tonsil, and the epithelial barrier in the crypts may only be one cell layer thick. The cryptal surfaces are described as “reticulated epithelium” to explain the arrangement in which epithelial cells, leukocytes and stromal cells are all juxtaposed in close proximity to the external environment [27]. In a morphological arrangement that resembles cryptal surfaces, there is only a single layer of columnar epithelial cells protecting the endocervical surface and leukocytes are commonly detected in focal cell clusters immediately beneath the endocervical surface. There is however, one significant difference between the cryptal and endocervical surfaces that relates to the secretion of mucus. Epithelial cells that constitute the endocervical surface continuously produce protective mucus, creating a physical barrier to prevent direct microbial contact with the epithelial surface. The viscosity of the mucus and the actual quantity of mucus produced are both regulated by hormonal signals in the female reproductive tract and it has been recognized that susceptibility to microbial infections can vary in relation to the timing of the menstrual cycle [28].

## Results and Discussion

**Figure 1:**
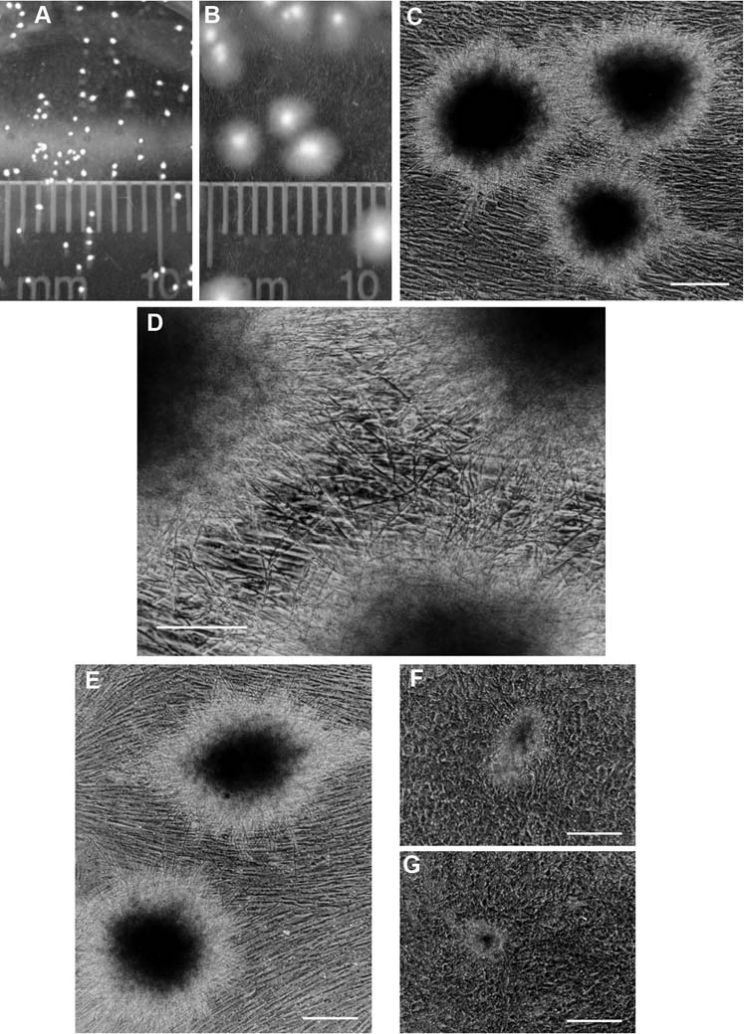
*C. albicans* colony formation on confluent monolayers of primary human cells. A) Direct observation of *C. albicans* colonies after 24 hours of coculture on primary tonsillar fibroblasts. B) Direct observation of *C. albicans* colonies after 48 hours of coculture on primary tonsillar fibroblasts - the same cell populations as panel A photographed 24 hours later. C) *C. albicans* colonies after 24 hours of coculture on primary tonsillar fibroblasts viewed by phase contrast microscopy. Bar = 200 µm. D) Magnified phase contrast image from (C) showing the central area between the 3 independent *C. albicans* colonies. Bar = 100 µm. E) *C. albicans* colonies after 24 hours of coculture on primary cervical fibroblasts viewed by phase contrast microscopy. Bar = 200 µm. F, G) *C. albicans* colonies after 24 hours of coculture on primary cervical epithelial cells viewed by phase contrast microscopy. Bar = 200 µm. Note that the colonies shown in panels E–G were cultured and photographed under identical conditions.

Many Candida*-*epithelial cell coculture experiments have been performed that involve large numbers of *C. albicans* cells causing rapid destruction of monolayers of immortalized epithelial cells. In the context of a natural infection, however, colonization of a mucosal surface or the solid surface of an in-dwelling device is most likely to involve *C. albicans* outgrowth from a low initial cell density [29]. We have therefore examined conditions that allow the formation of individual colonies from single *C. albicans* cells that have attached to monolayers of primary human cells, using inocula in the range of 50–250 cells/ml (Fig 1 A–D). We noted that there were differences in both the efficiency of colony formation and the size and complexity of the resultant colonies that were formed on the surfaces of various primary human cell populations (Fig 1 E–G). In all cases, there was minimal evidence for degradation of the mammalian cells during the first 48 hours of coculture. Continuation of cocultures beyond 48 hours did result in extensive human cell cytotoxicity but we have not investigated whether this cytotoxicity is a direct or indirect consequence of the *C. albicans*.

*C. albicans* colony formation readily occurred on fibroblasts derived from either tonsil or cervix. These primary fibroblast populations were characterized by filamentous or spindle cell morphology and the cells expressed high levels of vimentin [30]. In contrast, *C. albicans* colony formation was less efficient on primary epithelial cell populations with cuboidal morphology and prominent cytokeratin expression [24]. Colonies that formed on epithelial cells were reduced in terms of both colony size and number (Fig 1 E–G). In several cases, mixed fibroblast/epithelial primary cell populations expanded concurrently in adjacent but non-overlapping zones on the same transwell membrane and preferential attachment of *C. albicans* colonies to fibroblasts was consistently observed. This finding suggests that the impairment to *C. albicans* colony formation on epithelial cells was not due to release of soluble inhibitory factors but instead indicated a process that was disruptive to primary *C. albicans* attachment or growth. In this context, it is important to emphasize that the primary cervical cell populations did not produce any of the viscous mucus characteristically formed when endocervical tissue pieces were maintained in organ culture [25]. It is conceivable that the primary epithelial cells were deficient in one or more cell surface macromolecules required for efficient *C. albicans* binding and subsequent colony development. It is also conceivable that a very high surface concentration of anti-Candida proteins expressed and released by epithelial cells may be contributing to this growth difference [31], [32], [33] but there was no generalized inhibition of *C. albicans* growth in the cocultures, in the absence of direct contact with epithelial cells. These primary mixed cell culture systems can therefore be used as a readily accessible model to study human host responses to *C. albicans* [34], [35].

In the course of expanding primary fibroblast populations from fresh human tissue, we have been able to manipulate the culture conditions to achieve simultaneous outgrowth of both primary human fibroblasts and expansion of primary human Candida isolates from the same starting tissue (N = 8: 7 tonsils and 1 cervix; Table 1). Candida proliferation was limited, but not totally eliminated in the presence of amphotericin B, and primary human fibroblast proliferation, from both tonsil and cervix was largely unaffected by the low cell numbers of Candida. The amphotericin B allowed an extended interaction to develop between the mammalian and fungal cells, over a period of several weeks that could be considered as resembling the initial stages of colonization, and long-term infection at low levels on a mucosal surface. These observations also reinforce the idea that natural populations of Candida may not be completely eradicated by “cidal” antifungals *in vivo* and may remain as reservoirs from which drug resistant variants may emerge [36]. It is also noteworthy that 4/7 Candida isolates from palatine tonsil were obtained from children, 2–3 years old. These children were undergoing routine tonsillectomy with no evidence of widespread oral Candida infection at the time of surgery. Several recent studies have examined Candida infections in neonates [37], [38], [39] but there is seemingly less information available relating to the presence and isolation of Candida from very young children [40]. The samples processed in this study provide direct evidence for oropharyngeal Candida colonization in 2–3 year-old children.

**Table 1:** Matched Primary Patient Isolates: Candida Strains and Proliferating Human Cell Populations.

Patient/Age	Tissue	Clinical Condition/Histology	Candida Species
Male/68	Tonsil	Not Available	*C. albicans*
Female/18	Tonsil	Chronic Tonsillitis/Hyperplasia	*C. glabrata*
Female/2	Tonsil	Sleep Apnea/Hyperplasia	*C. albicans*
Female/16	Tonsil	Chronic Sore Throat/Hyperplasia	*C. albicans*
Female/3	Tonsil	Streptococcal Tonsillitis/Hyperplasia	*C. albicans*
Female/2	Tonsil	Not Available	*C. albicans*
Male/3	Tonsil	Snoring	*C. albicans*
Female/36	Cervix	Endometriosis/Mild Cervicitis	*C. glabrata*

**Figure 2:**
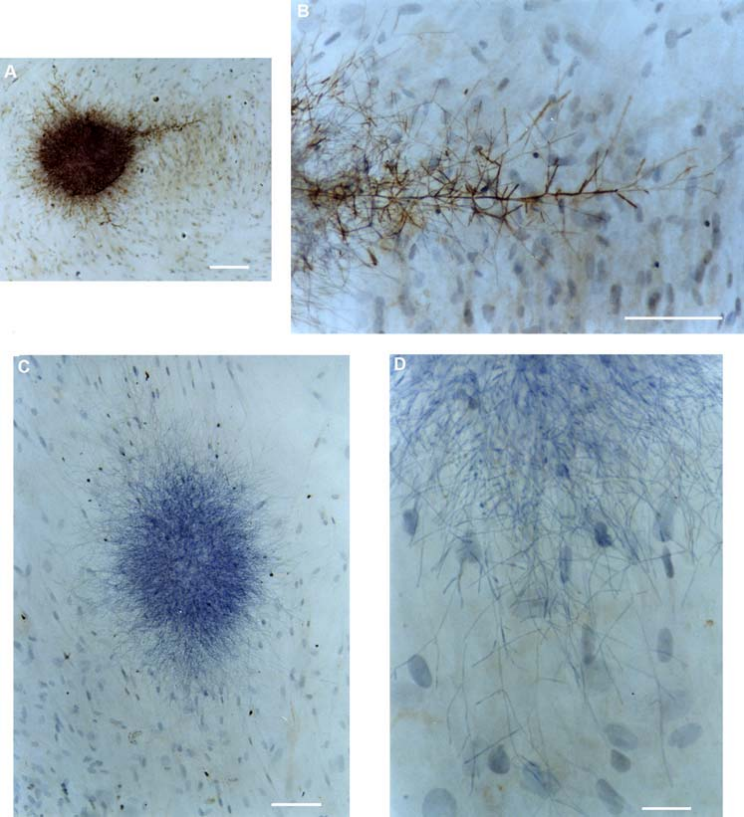
Analysis of filamentous extensions from *C. albicans* colonies growing on human cells. A) Immunocytochemical staining with a rabbit polyclonal antibody of a *C. albicans* colony grown on top of a monolayer of primary human tonsillar fibroblasts. The human cells were counterstained with hematoxylin. Bar = 200 µm. B) Immunocytochemical staining of the same *C. albicans* colony in panel A, showing a major filament extending across the surface of the primary tonsillar monolayer. Bar = 100 µm. C) Hematoxylin staining of a *C. albicans* colony growing on top of a monolayer of primary human tonsillar fibroblasts. The mammalian cell nuclei are visible directly beneath the colony structure. Bar = 100 µm.D) Enlarged image of the lower extremities of the colony shown in panel C. Bar = 25 µm.

**Figure 3:**
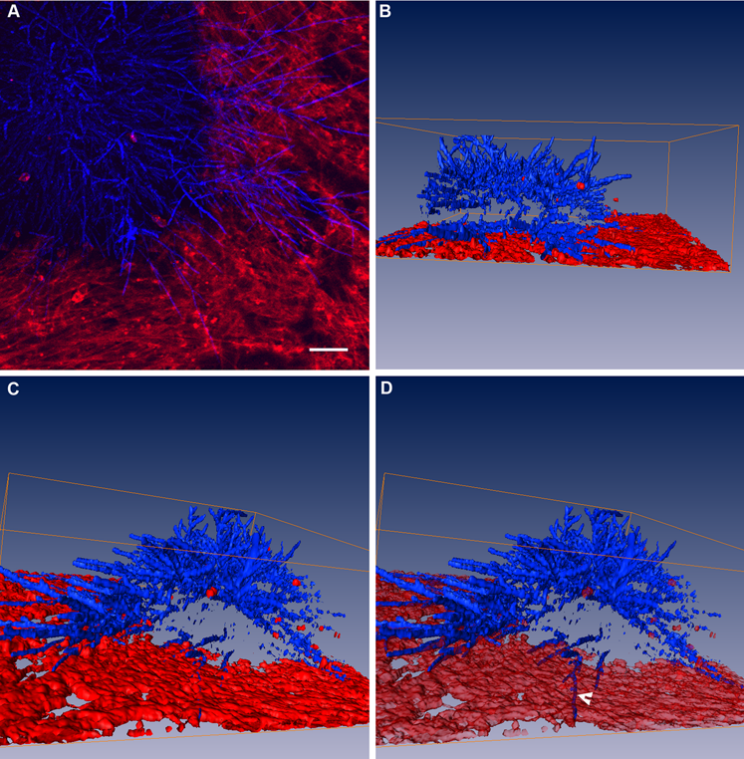
Confocal microscopy and three-dimensional image reconstructions of a *C. albicans* colony growing on human cells. A) Brightest point projection of a confocal Z series showing a *C. albicans* colony (stained blue with calcofluor) grown for 22 hours on a monolayer of primary human fibroblasts (stained red with wheat germ agglutinin). Bar = 100 µm. B) Three-dimensional reconstruction of the colony shown in panel A. This image has been rotated relative to the image shown in panel A. C) Different perspective of the three-dimensional reconstructed image shown in panel B. D) Exactly the same reconstructed view of the colony as shown in panel C but with the surface of the fibroblast monolayer now rendered semi-transparent to reveal fungal cells beneath the mammalian cell surface. Note the continuous appearance of the filamentous extension (arrowhead in D) that is not visible with the opaque monolayer surface in panel C.

We have developed a series of immunocytochemical and immunofluorescence detection procedures to visualize the structural features of Candida colonies attached to mammalian cells (Figs 2 and 3). One characteristic feature was the formation of complex filamentous structures, extending from the central mass of the Candida colonies that appeared when the mammalian cells were propagated on either transwell membranes or glass chamber slides (Fig 2 A–D). At present, it is unclear if these filamentous structures should be better regarded as “colony anchors”, as “exploratory sensors” or as supporting a combination of both functions. We have used fluorescent reagents – calcofluor staining of Candida cells and wheat germ agglutinin conjugated to Alexa Fluor-633 counterstaining of mammalian cells – to develop a rapid method of visualizing *C. albicans* filaments by confocal microscopy (Fig 3 A). Additionally, we have used the surface reconstruction program AMIRA to create, for the first time, a three-dimensional representation of invasion by *C. albicans* filaments (Fig 3 B). By adjusting the transparency of the reconstructed surface, it was possible to identify filamentous extensions from the *C. albicans* colonies that had penetrated below the surface of the mammalian cells (Fig 3 C, D).

One limitation with confocal microscopy arises from uncertainty regarding the efficiency of penetration of fluorescent dyes and antibodies in detecting specific targets in multi-layered cell structures and tissue pieces. For example, the absence of any detectable fluorescent signal may be explained either by the absence of target cells to generate a signal or by incomplete penetration of the labeling reagents. These limitations may be partially circumvented by using conventional immunocytochemical procedures to analyze both the Candida colony forms and the underlying mammalian cells (Fig 2 C, D). From the combination of immunocytochemical and immunfluorescence labeling procedures and direct observations by phase contrast microscopy, it appeared that the there was minimal disturbance to the mammalian cells situated directly beneath the *C. albicans* colonies. It is also readily apparent that the overall cell density for Candida colonies formed in liquid medium is much less than the cell density for colonies grown on conventional nutrient agar plates.

**Figure 4:**
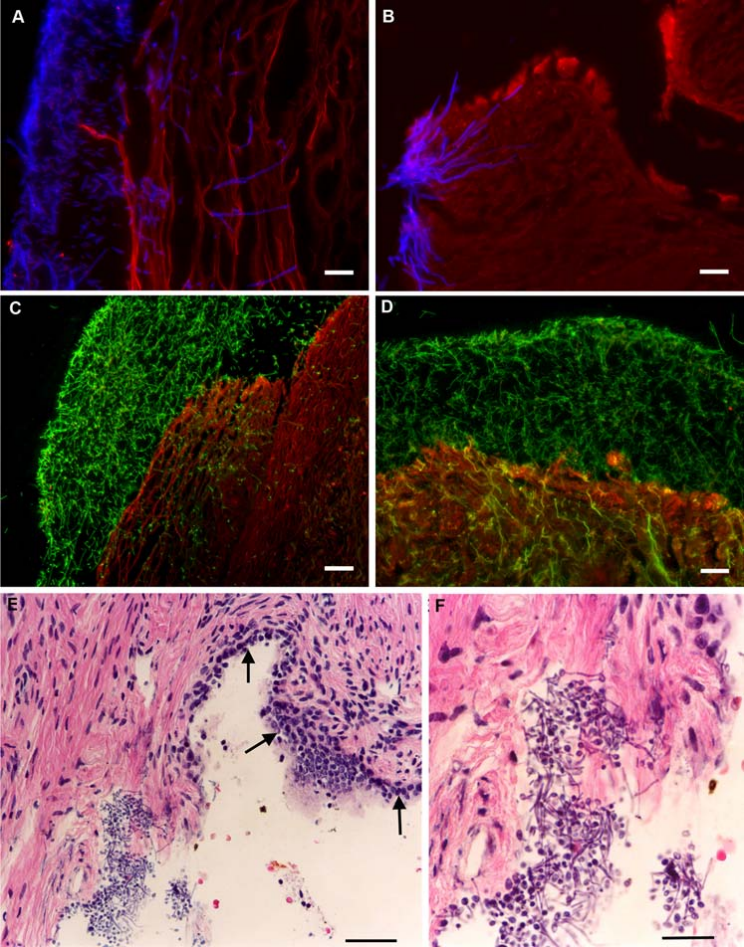
*C. albicans* invasion of human tissue in *ex vivo* organ culture. Freshly removed human tissue pieces were cocultured with *C. albicans* for 24 hours and then the tissue pieces were fixed in formalin and embedded in paraffin. 5 micron sections were stained with fluorescently labeled reagents and visualized by fluorescence microscopy (A–D) or stained with hematoxylin and eosin and viewed by standard bright field microscopy (E, F). These images are representative of experimental *C. albicans* colonization and invasion for five different premenopausal human cervical tissue pieces. A) *C. albicans* yeast cells and filaments (stained blue with calcofluor) invading the stratified squamous epithelium of human ectocervix (stained red with wheat germ agglutinin). Bar = 25 µm. B) *C. albicans* filaments (blue) invading the cut stromal surface of human ectocervix (red). Bar = 25 µm. C) *C. albicans* yeast cells and filaments (green: detected with a rabbit polyclonal anti-*C. albicans* antibody conjugated to FITC) invading the stratified squamous epithelium of human ectocervix (stained red with wheat germ agglutinin). Bar = 50 µm. D) *C. albicans* yeast cells and filaments (green: detected with a rabbit polyclonal anti-*C. albicans* antibody conjugated to FITC) invading a cut stromal surface of human ectocervix (stained red with wheat germ agglutinin). Bar = 50 µm. E) *C. albicans* growth on the cut stromal surface of endocervix. Note the absence of *C. albicans* growth on the columnar epithelial surface of endocervix (arrows). Bar = 50 µm. F) Magnified image of (D) showing colonization of the cut stromal surface of endocervix. Bar = 25 µm.

We have also explored the process of Candida colony formation on the surface of tissue pieces by placing freshly isolated mucosal tissue into proliferating *C. albicans* cultures. With premenopausal cervical tissue, microscopic colonization and invasion occurred readily on the stratified squamous epithelial surface of the ectocervix and on cut surfaces of cervical tissue where *C. albicans* had direct access to stromal tissue (Fig 4 A–D). Conversely, the columnar epithelial surface of the endocervix appeared refractory to *C. albicans* colonization whereas cut stromal surfaces immediately adjacent to columnar epithelial cells readily supported *C. albicans* outgrowth (Fig 4 E, F). We have consistently observed the production of copious amounts of mucus by endocervical epithelial cells, under different organ culture conditions and the presence of mucus, together with the protective mechanism disrupting colony formation on primary cervical epithelial cell populations may account for the inability to colonize the columnar epithelial surface of the endocervix.

**Figure 5:**
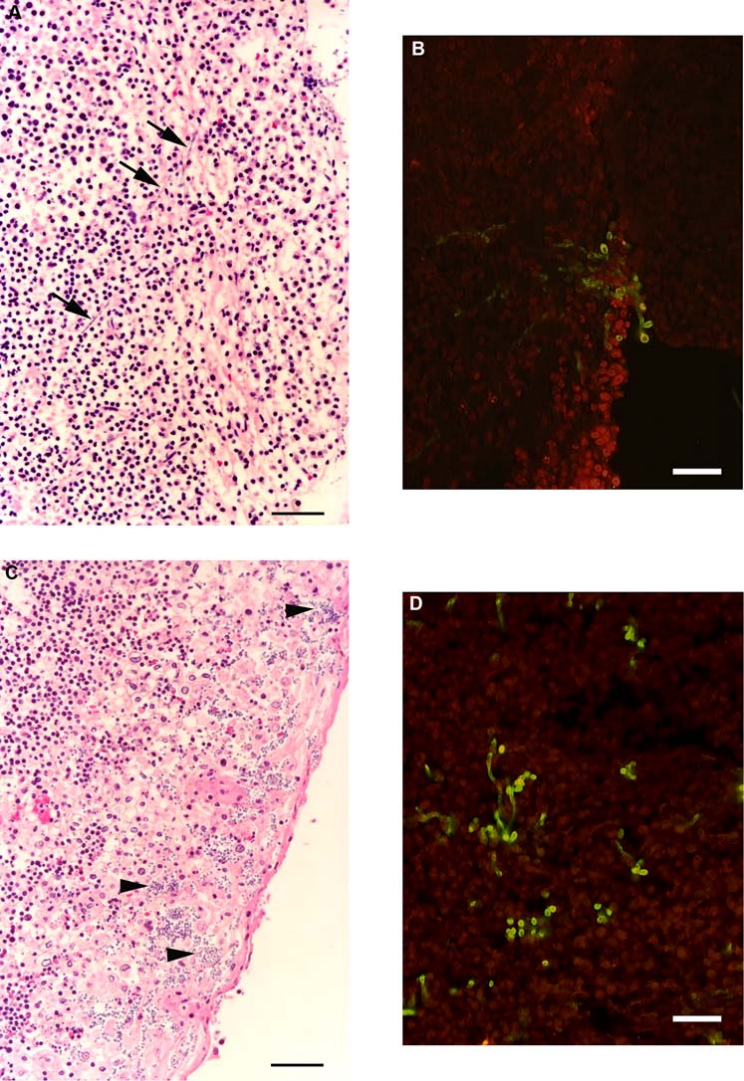
*C. albicans* outgrowth from pre-existing natural infections during *ex vivo* organ culture with human palatine tonsil. Freshly removed pieces of human palatine tonsil were cultured for 4 days in complete RPMI medium containing antibiotics and amphotericin B, fixed and processed for routine histological analysis. A, C) Hematoxylin and eosin staining of 5 micron tissue sections showing filaments (arrows) and yeast cells (arrow heads) at or near the luminal surfaces of two different palatine tonsil tissue pieces. Tonsil A: 23 year old male patient with recurrent tonsillitis. Tonsil B: 13 year old female patient with snoring problems. Bars = 50 µm. B, D) Merged fluorescence images of 5 micron sections from the corresponding tissue blocks shown in A and C respectively that were incubated with a rabbit polyclonal anti-*C. albicans* antibody conjugated to FITC (green) and then the tissues were counterstained with wheat germ agglutinin (red). Bars = 50 µm.

During the normal course of *ex vivo* organ culture experiments with human palatine tonsil, we have occasionally noticed the appearance of “fungal-like” cell bodies in fixed tissue samples that had been sectioned and stained with hematoxylin and eosin (Fig 5 A, C). These tissue pieces had been incubated for four days in complete RPMI medium containing antibiotics and amphotericin B. We believe that the fungal forms represent limited outgrowth from pre-existing infections. Unfortunately, there is no mechanism to recover viable isolates from these fixed organ culture samples but we have been able to establish the presence of Candida by specific staining with a rabbit polyclonal antibody (Fig 5 B, D). In the context of these natural colonization events, yeast cells were observed more commonly than filamentous structures, probably due to the presence of amphotericin B in the culture medium. Both tissue pieces have diffuse inflammatory cell infiltrates in proximity to the fungal forms but there was no specific recruitment of neutrophils, as would be expected with equivalent fungal infections of mucosal surfaces in immunocompetent individuals (Fig 5 A, C). The absence of focal inflammatory cell responses can be explained by the lack of any blood supply to the tissue pieces in these *ex vivo* organ cultures.

The distinctions that were observed between *C. albicans* colonization of epithelial cells, fibroblasts and tissue surfaces were quite striking and may have important biological implications. The apparent ease of colonization of the external layers of stratified epithelium could be offset by the fact that the most external cells are continuously lost by sloughing and therefore Candida invasion through intact stratified squamous epithelium may only occur at low probability. The combined results with endocervix and the primary cervical epithelial cells suggest that there may be multiple mechanisms acting in concert, including mucus secretion by endocervical epithelial cells that confer protection against Candida colonization. Conversely, the efficient experimental colonization that we have observed with stromal cell surfaces exposed on cut tissue pieces and with primary fibroblasts can be projected to occur either when the protective epithelium has been damaged and/or when repair processes have been activated after surface tissue damage has occurred. Micro lesions are commonly observed in the reproductive tract of sexually active young women [41] and fibroblasts are widely distributed immediately below normal epithelial surfaces (PS unpublished results for premenopausal human cervix; see [30], [42] for human palatine tonsil). The likelihood of progressive damage at mucosal surfaces, or imperfect repair after damage is therefore increased substantially when epithelial integrity has been disrupted for the first time and this may be an important contributory factor to recurrent vaginal candidiasis that is experienced by some young women. A direct connection between primary infection and increased susceptibility to subsequent infections has been suggested from several independent studies of microbial infections at mucosal surfaces and is now widely accepted as a significant co-factor in community-acquired infections [43], [44], [45].

As discussed and demonstrated here for *C. albicans*, and by the work of others with widely divergent microbes including *T. vaginalis* [46], *Mycoplasma* spp., *C. trachomatis* and *N. gonorrhoeae* [47], [48] and Kaposi's Sarcoma Herpes Virus [49], primary human cell systems and *ex vivo* organ culture methods can now be considered as a robust experimental platform for current and future studies of microbial infections at human mucosal surfaces (for comprehensive reviews see [50], [51], [52]). The relative merits of individual *ex vivo* experimental systems are still very much under evaluation because different experimental questions will probably emphasize a different weighting between the critical parameters of convenience, reproducibility and connection to biological reality. For example, in both the oropharynx and the premenopausal female reproductive tract, not all mucosal surfaces are keratinized so the presence or absence of surface keratin in a reconstituted epithelium becomes an important detail of overall experimental design. Systems with a component of immortalized cells score highly for convenience and reproducibility but may be somewhat lacking in the biological exactness of the reconstructed epithelial surface, especially with the use of heterologous cell constituents [52]. Furthermore, it is becoming increasingly clear that structural variability is a normal feature of mucosal surfaces [25], [42] and that mucosal surface variability may well be a significant factor in distinguishing between the outcomes of exposure without infection, sub-clinical infection or clinical infection. The experiments we have conducted with surgically removed tissue samples represent a meaningful approximation to the natural processes of exposure and infection in the patient. However, the availability of tissue is unpredictable and every tissue piece is different. As a consequence, although the *ex vivo* organ culture experiments might be preferred for overall biological relevance, the organ culture systems create some challenges for convenience and reproducibility. The organ cultures also provide the opportunity to examine polymicrobial systems by virtue of the presence of mucosal commensal organisms and any pathogens present on the surface or within the tissue pieces. The results shown in Table 1 and Figure 5 fully illustrate this process as tissue pieces disrupted for primary cell expansion or prepared for experimental HIV infection also supported outgrowth of candida from low-level pre-existing mucosal infections.

The experiments with primary cell populations are at least one step further removed from reality than tissue pieces in organ culture but primary cells do provide biological value relative to standard immortalized epithelial cell lines. As culture conditions become better defined, either to support the specific expansion of homogeneous primary cell populations or to support the survival and expansion of complex primary cell mixtures derived from disrupted mucosal surfaces, so the utility of primary cell populations should increase. Currently, we are routinely using primary cell populations to validate new questions and reagents that subsequently will be addressed in organ culture systems. Furthermore, on a practical level, because not all tissue pieces are of sufficient quality for organ culture experiments we have found that tissue pieces with limited surface epithelium can still be put to good use in the derivation of primary cell populations [24], [42].

The individual and collective benefits of reconstituted human epithelial surfaces, *ex vivo* organ cultures and primary human cell populations will continue to be recognized as more investigators begin to exploit these experimental systems. Progress to date reflects technical insight and innovation in many different laboratories. Most significantly, the resources and procedures are now available to support fundamental studies of human microbial infection and pathogenesis using biologically relevant human mucosal surfaces.

## Methods

### Tissue samples and derivation of primary cell populations

Human tissue samples were obtained within 1–2 hours of the completion of surgery from the Tissue Procurement Facility maintained within the University of Minnesota Hospital, Fairview. Methods describing the manipulation of tissue pieces on collagen supports and the preparation of primary cell populations on transwell membranes (Cell Culture Inserts [0.4 µm pore size, polyethylene tetraphthalate membranes without additional ECM coating] BD Biosciences, Bedford MA) have already been published [24], [42]. The experimental protocols used here had full IRB approval (Institutional Review Board: Human Subjects Committee, Research Subjects' Protection Program, University of Minnesota) and informed written consent was obtained from individual patients, or the legal guardians of minors, for the use of tissue in research applications prior to the initiation of surgery. With most tissue samples, part of the tonsil was immediately fixed in Streck Tissue Fixative (STF; Streck Laboratories, La Vista , NE) and then processed for paraffin embedding, sectioning and hematoxylin and eosin (H&E) staining. The tissue sections revealed extensive hyperplasia and tonsillar fibrosis, as would be anticipated in patients requiring tonsillectomy, but no major histopathological abnormalities. Foci of bacteria were observed at and just beneath the external tissue surface and within the tonsillar crypts but no yeast cells or filaments were observed in the H&E sections. Cocultures of tissue pieces with *C. albicans* were set up in RPMI medium containing 10% percent heat-inactivated fetal calf serum but lacking antibiotics or amphotericin B. Primary cell populations were derived in RPMI medium containing 10% percent heat-inactivated fetal calf serum with antibiotics and amphotericin B (200units/ml penicillin, 200units/ml streptomycin, 500ng/ml amphotericin B [Antibiotic-Antimycotic #15240-062; Invitrogen, Carlsbad CA]) and then the cells were cultured overnight in medium lacking amphotericin B, prior to the addition of viable *C. albicans*.

### Immunocytochemistry and Immunofluorescence

For fluorescence detection, Candida were visualized using calcofluor to stain components of the cell wall or by staining with a rabbit polyclonal anti-*C. albicans* antibody that was directly conjugated to FITC (Catalog # B65411F; Biodesign International, Saco, ME). Tissue sections or primary human cell populations on transwell membranes were counterstained with wheatgerm agglutinin (Alexa Fluor-633 conjugate; Molecular Probes, Eugene, OR).

### Confocal Microscopy

Primary data sets were collected using a Bio-Rad 1024 Laser Scanning Confocal microscope (Bio-Rad: Hercules, CA) equipped with a krypton/argon laser or a two photon system using krypton-argon and mercury vapor lasers. Images were processed using Confocal Assistant, Image J, AMIRA and Volocity software programs.

### Candida

Cells from overnight cultures of DAY185 [53] were washed and resuspended in HBSS. Serial dilutions were prepared and appropriate volumes of *C. albicans* were added to the upper surfaces of transwell membranes that contained primary populations of fibroblasts and epithelial cells and cultured in RPMI medium containing 10% percent heat-inactivated fetal calf serum but lacking antibiotics or amphotericin B. The cocultures were then incubated at 37C in standard CO2 tissue culture incubator (5% CO2) for 24–48 hours. Primary patient isolates were initially propagated in tissue culture medium and then samples were streaked on YPD plates (1% yeast extract, 2% bacto-peptone, 2% dextrose 80 µg/ml uridine and 2% bacto-agar). Individual colonies were then expanded and stored. Candida typing was based on germ-tube formation and restriction enzyme digestion patterns [54].
